# Microbial pathogens of edible insects: a growing problem for the insect farming industry

**DOI:** 10.1042/ETLS20253013

**Published:** 2025-12-09

**Authors:** Stephen E. Akemu, Alexandra E.G. Welford, Roger D. Santer, David E. Whitworth

**Affiliations:** 1 Department of Life Sciences, Aberystwyth University, Aberystwyth, Ceredigion, SY23 3DD, U.K

**Keywords:** disease transmission, entomopathogens, epizootic disease, farmed insects, food safety, probiotics

## Abstract

Insect farming is widely extolled as a sustainable alternative to the traditional agricultural production of protein for human and animal consumption. However, pathogen contamination endangers insect health, food safety, production yields and market acceptance. Because insect farming is intensive, growth and transmission of pathogens are promoted, elevating the risk of disease outbreaks with severe economic outcomes. Fungal pathogens can invade host insects through their cuticle, reproducing within the nutrientrich haemolymph within the haemocoel until the host’s defences are overwhelmed and the insect dies. Other pathogens, such as viruses, oomycetes and bacteria, enter the host orally before penetrating the midgut wall to infect the haemolymph. Even apparently healthy farmed insects carry a diverse array of potentially pathogenic bacteria/fungi within their guts, as well as sub-lethal viral infections, and these covert infections can quickly become epizootic breakouts. Consequently, there is an urgent need to understand the infection and transmission of pathogens in insect farms, as well as to develop strategies to prevent and treat infections/outbreaks.

This review collates information about the susceptibility of farmed insects to infection by fungi, bacteria, viruses, nematodes and other parasites, current pathogen detection methods, and possible control measures, with the aim of making this information accessible to practitioners and researchers of insect farming. We suggest that prophylactics/treatments are urgently needed by insect farms, alongside improvements in infection control, to ensure the long-term viability and acceptance of edible insects as a sustainable alternative protein source.

## Insects for sustainable protein production

By 2050, the world’s population is predicted to reach approximately 10 billion people, with an estimated 150 million at risk of dietary protein deficiency [1]. The Food and Agriculture Organisation of the United Nations estimates that worldwide protein production needs to increase by 76% to meet growing demand [2], putting considerable pressure on our agricultural systems to provide more high-quality, sustainable protein.

Currently, livestock-derived protein production accounts for 18% of all global carbon emissions and utilises around 70% of global agricultural land, with harmful environmental impacts via soil degradation, loss of biodiversity, greenhouse gas emissions, water consumption and pollution [2,3].

Edible insects are increasingly recognised as an environmentally friendly and sustainable source of dietary protein and have the potential to support our future food security [4]. Insect protein production has less environmental impact than traditional livestock farming, with increased feed conversion ratios, reduced land and water use (e.g. mealworms produce 1 g of protein per 23 l of water compared with 112 l for beef), and a negligible contribution to global warming compared with traditional agriculture [3,5,6]. Nutritionally, insects are high in protein (50–82%), fat, vitamins, minerals and fibre, although the nutritional profile of insects is highly variable across species and can even vary within species depending on factors such as diet, metamorphic stage, habitat and environmental conditions [5,7].

The environmental benefits of insect farming are made possible because it is intensive, which inevitably puts insect farms at risk of disease outbreaks. Aimed at both insect farmers and researchers, this review provides an overview of insect pathogens, their routes of transmission and their threat to insect farms. It also identifies strategies for the prevention, diagnosis and treatment of outbreaks – highlighting current gaps in knowledge and suggesting potential treatments for targeting microbial disease.

### The increasing scale of edible insect farming

While the consumption of insects (entomophagy) has been practised throughout human history and remains commonplace in various African, Asian and South American cultures, the insects consumed are often obtained through ‘wild harvesting’ rather than deliberate cultivation [8,9]. Globally, the large-scale rearing of insects for human consumption is relatively uncommon, mainly because negative public perceptions and lobbying in Western cultures have relegated edible insects to a novelty food source or as a source of feed for livestock and not as a routine part of the diet [10,11].

**Figure 1:**
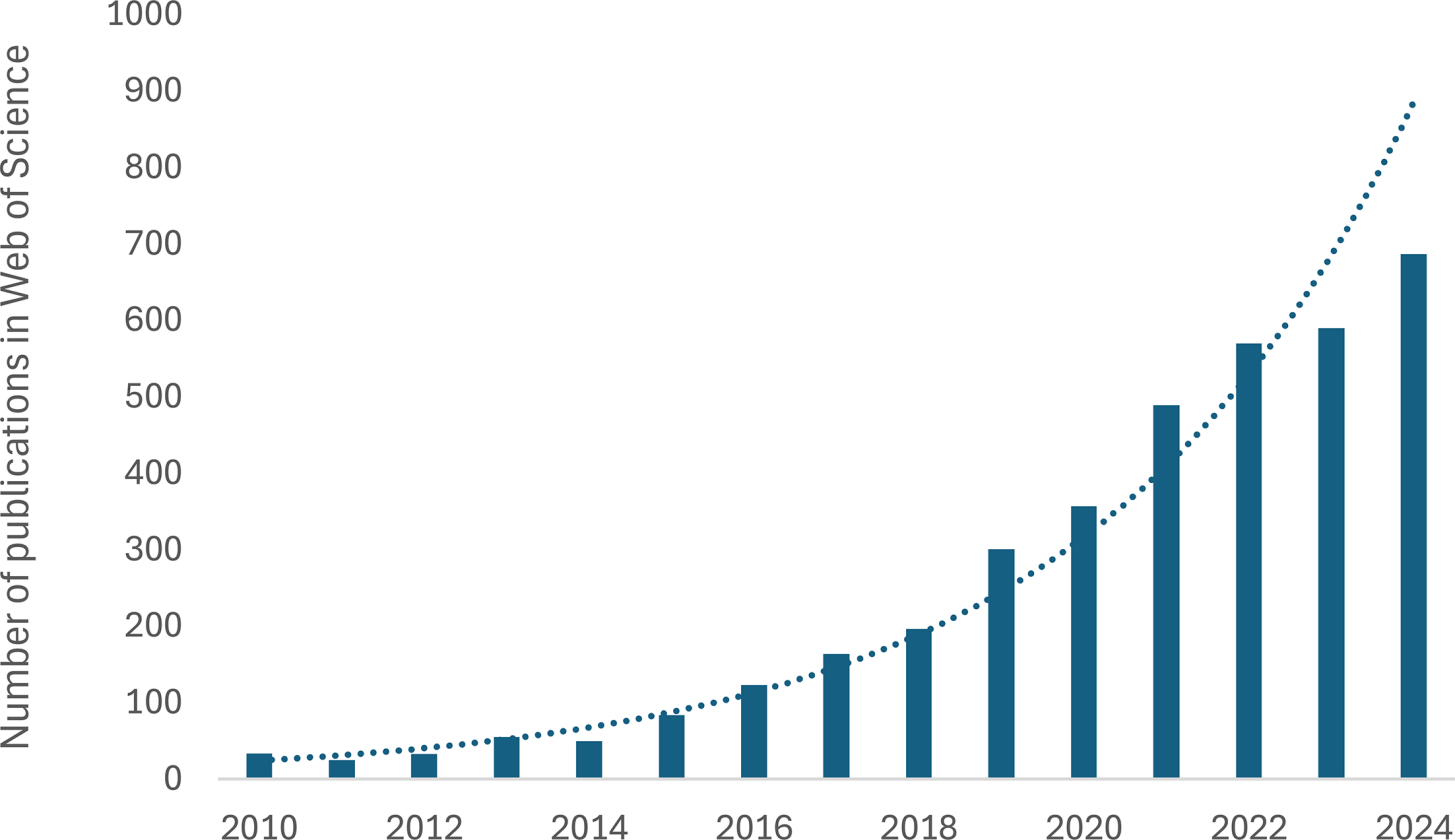
Edible insect farming publications. Publications retrieved from Web of Science using the search ‘soldier fly farm* OR cricket farm* OR edible insect’ as a function of year published (search performed 3rd July 2025). The trendline shows a CAGR of 26% starting from 34 publications in 2010.

In response to their promise as a sustainable protein source, the edible insect farming sector has grown exponentially in recent years and is predicted to continue doing so, with compound annual growth rates (CAGRs) typically estimated to be around 25%. One typical such forecast based on the global edible insect sector being worth over US$1.35 billion in 2024, predicts the market will exceed US$4.4 billion by 2030 [12]. The exponential growth of the sector over the last 10–15 years has been mirrored by publications in the scientific literature on edible insects and insect farming, with a similar CAGR (Figure 1).

With insects increasingly recognised as an important component of the global food system, legislative focus has begun to enforce stricter production requirements [13]. In 2015, the European Union (EU) classified edible insects as a novel food, leading to a temporary ban on insect products and a requirement for insects as food to go through an authorisation process [14]. Some countries (including the UK, Netherlands, Belgium, Denmark and Finland) continued production, arguing that animal products do not require novel food authorisation. However, in 2015, the EU specified that from 2018 all insect producers must receive authorisation to market their products [15].

From January 2024, food products containing edible insects could only remain on the market in the United Kingdom if the Food Standards Agency or Food Standards Scotland had received a novel food application [16]. Applications for four insects were received by December 2023: yellow mealworm (*Tenebrio molitor,* Coleoptera), house cricket (*Acheta domesticus*, Orthoptera), banded cricket (*Gryllodes sigillatus*, Orthoptera), and black soldier fly (*Hermetia illucens*, Diptera). Transitional regulations had previously also allowed the rearing of desert locust (*Schistocerca gregaria*, Orthoptera), migratory locust (*Locusta migratoria*, Orthoptera) and lesser mealworm (*Alphitobius diaperinus*, Coleoptera) [16].

In the UK, the farming of insects for animal feed rather than human food is regulated by agencies such as the Department for Environment, Food and Rural Affairs, the Animal and Plant Health Agency and the Environment Agency. Under their collective auspices, seven insect species are farmed in the UK – the four insect species farmed for food listed above, plus the common housefly (*Musca domestica*, Diptera), lesser mealworm and Jamaican field cricket (*Gryllus assimilis*, Orthoptera) [17].

There are economic benefits to growing insects on cheap waste streams from crop and animal production; for instance, black soldier fly larvae can be grown on manure (albeit for feed and not human consumption); however, this comes with the associated risk/certainty of contaminating the insects with potentially pathogenic microbes [18,19].

This, combined with the density at which insects are kept, the increasing scale of insect farming and a comparative lack of guidelines/regulations and veterinary knowledge to support their health and welfare, suggests a great need to understand diseases of insects in captivity and develop protocols for managing them.

## Entomopathogen threats to farmed insects

A significant obstacle to the sustainable production of edible insects and their acceptance by consumers is disease, which can reduce farmed insect survival, food safety and protein yield. Many entomopathogens (infectious microorganisms which cause disease in insects) are opportunistic and have a broad host range, making them widespread and easily transmitted [20]. Pathogen accumulation and persistence in high-density insect rearing systems can cause both horizontal and vertical transmission cycles, increasing the risk of epidemics [21]. Indeed, entomopathogens are known to cause disease outbreaks in insect rearing systems, resulting in high mortality with severe economic losses [22,23]. For instance, *A. domesticus* densovirus (AdDV) has caused disease outbreaks in farmed crickets, resulting in the bankruptcy of cricket-rearing firms [24]. In addition to die-offs, infections can reduce insect feeding, reproduction and growth rates [25]. However, there is a comparative lack of detailed knowledge on this issue.

To date, entomopathogens have primarily been investigated and exploited from a crop protection point of view, with a focus on reducing rather than safeguarding insect populations. For example, the entomopathogenic fungi *Beauveria bassiana* and *Metarhizium* spp., the bacterium *Bacillus thuringiensis* and viruses including *Helicoverpa zea*/*Spodoptera exigua* nuclear polyhedrosis viruses and granulosis virus of *Cydia pomonella* have been commercialised for insect pest management in agricultural production and disease vector control [26,27]. Insects have also been used extensively as models for investigating immune responses to pathogen infection [25]. Consequently, we know a lot about the microbes which infect insects and how they do so, although we know relatively little about their abundance and spread in the context of insect farms. Table 1 provides examples illustrating the wide range of entomopathogens known to infect edible insects farmed for food/feed.

**Table 1:** An illustrative selection of farmed insect pathogens, their mechanisms of action and pathology

Pathogen type	Pathogen	Mechanism of action	References
Bacterium	*Serratia marcescens*	Penetration of the insect hemocoel after ingestion. Toxin secretion causes tissue damage and organ failure	[28,29]
Bacterium	*Escherichia coli*	Microbes in the haemolymph stimulate the immune system of individuals surviving initial bacteraemia.	[30]
Fungus	*Metarhizium anisopliae*	Fungi infect insects through the host cuticle or by ingestion, causing dysbiosis of the gut microbiome.	[31,32]
Fungus	*Beauveria bassiana*	Conidia/spores attach to the cuticle via hydrophobic interactions, penetrate it and enter the hemocoel. Can also cause pathology if ingested.	[31,33]
Fungus (microsporidian)	*Nosema grylli*, *Antonospora locustae*.	Obligate parasite. Invasion of epithelial cells after consumption of spores via a ‘polar tube’.	[34–36]
Virus	*Acheta domesticus* densovirus (AdDV)	Virus particles are consumed from food or faeces. The virus reaches the haemocoel, where it replicates in the nuclei of rapidly proliferating cells, spreading through the haemolymph.	[37,38]
Virus	Cricket iridovirus (CrIV)	Present in populations as covert infections. The virus is transmitted horizontally via the mouth and potentially also vertically.	[37]
Protozoan	*Malpighamoeba* spp.	Infected malpighian tubules are filled with amoebal cysts, have thinner walls and slower rhythmic movements.	[39]
Parasite (nematode)	*Steinernema spp*.	Infective juveniles enter the haemocoel via natural holes (moth/anus/spiracles). Once inside the host, they release symbiotic bacteria carried in their digestive tract, which kill the insect via septicaemia roughly 48 hours after infection.	[40–42]

### Fungi

Fungal entomopathogens like *Metarhizium anisopliae* and *B. bassiana* produce infectious conidia/spores as part of their life cycles. Dispersed spores germinate on a host insect’s cuticle, creating a germ tube which penetrates through the cuticle using mechanical pressure and enzymatic digestion [43]. The fungus then colonises the insect’s body, growing inside the host and eventually killing the insect and overgrowing its cadaver in fungal mycelium and conidia.

Microsporidia are obligate intracellular parasites, originally thought to be protozoans, though now recognised to be fungi. They mostly infect insects, including bees as well as edible crickets and locusts [44]. Consumed spores enter host gut epithelial cells by either extruding a polar tube into the cell or by phagocytosis, using the polar tube to escape the lysosome [34]. Infection can be lethal to insects, with symptoms of infection including impaired reproductive performance, movement and growth [44].

### Oomycetes

Oomycetes, which until recently were thought to be fungi, are predominantly pathogens of plants. However, some species (e.g. *Pythium guiyanense*) have evolved to infect mosquito larvae, and the emergence of further oomycete entomopathogens is a potential risk to farmed insects [45].

### Bacteria

Ingested bacterial entomopathogens, such as *B. thuringiensis* and *Serratia marcescens*, produce toxins that promote penetration of the insect gut epithelium and invasion of the haemocoel (body cavity). The bacteria can then reproduce rapidly, fuelled by the nutrient-rich haemolymph within the haemocoel, evading the immune system and secreting further toxins. The resulting septicaemia leads to diminished feeding, tissue damage, organ failure and ultimately mortality [28,30].

### Viruses

Viruses such as AdDV and cricket iridovirus are ubiquitously found within farmed cricket populations as non-lethal covert infections which can be transmitted horizontally (and possibly vertically), while remaining capable of initiating outbreaks of active infection [24,37]. Upon ingestion, non-enveloped icosahedral virus particles containing single-stranded DNA (densoviruses) or enveloped particles in occlusion bodies containing double-stranded DNA (baculovirus) infect the epithelial cells lining the midgut via endocytosis, and virus replication occurs, resulting in cell lysis. Some of the symptoms of viral infection can include behavioural changes (e.g. hyperactivity), bodily liquefaction, as well as a high death rate [46]. While all cricket species seem to maintain covert infections of AdDV, only *A. domesticus* is killed by this virus, prompting suggestions to switch production to alternative edible crickets such as *G. sigillatus* [24]. However, the high mutation rate in densoviruses means that resistant species could easily become susceptible in the future.

### Amoebae

No amoebic diseases of licensed food/feed insects have been reported yet, but the protozoan *Malpighamoeba* spp. can infect the Malpighian tubules of a wide range of insects, including bees and the edible desert locust, *S. gregaria* [39]. Malpighian tubules are secretory and osmoregulatory organs. Infection with Malpighamoebae compromises Malpighian tubule activity, increases water loss and causes premature death [39].

### Parasites

While not microbes, it is worth noting that edible insects are also prone to microscopic parasites such as nematodes, helminths and other parasitic worms [40], in addition to macroscopic parasites such as mites and parasitic wasps [47]. Typically, endoparasites are introduced into the insect by adults or otherwise enter the haemocoel post-ingestion, where they then consume the insect from within or cause host death through the release of pathogenic bacteria into the haemolymph [48].

## The pathogenic microbiota of farmed insects

In contrast with the detailed knowledge of potential pathogens summarised above, much less is known about the pathogens that actually occur on insect farms. Farmed insects have diverse microbiomes, which include a wide range of pathogenic organisms, some of which could potentially cause disease in humans. For this reason, farmed insects will sometimes be separated from their food/frass for a day prior to harvest, giving them a chance to empty their gut contents before they are processed for food [19].

Microbiome studies have shown that there is considerable variation in bacterial load and community composition within edible insects, which is influenced by factors such as host species, temperature and feed quality [49]. While this variability limits the generalisation of findings, examples of contaminating bacteria particularly important to the food processing industry include aerobic and spore-forming bacteria such as *Bacillus* spp., *Clostridium* spp., *Escherichia coli*, *Listeria monocytogenes*, *Salmonella* spp., and *Staphylococcus* spp [49,50]. . Edible insects have also been proven to be carriers of antimicrobial resistance (AMR), a potential risk not only to humans but also to disease control within the rearing system [51,52].

While there is little evidence to suggest that entomopathogenic fungi are directly harmful to humans (see Tucker et al. [53] for a rare example), the microbiome of edible insects can nevertheless be harmful for human consumption. For example, farmed insects can become contaminated with mycotoxins, which are poisonous to humans and other mammals [54]. Similarly, although entomopathogenic parasites cannot complete their life cycles within humans, there is some evidence that edible insects can act as intermediate hosts for several parasites, including tapeworms [49,52,55].

Given the taxonomic distance between insects and humans, the risk of transmitting or amplifying fungal, viral and parasitic zoonotic diseases is considered low [50]. However, intensive rearing of vertebrate livestock can cause extensive loss of animals through zoonotic disease, exacerbated by AMR [5]. We anticipate that similar dynamics may occur in insect farming, underscoring the need for further targeted research in this area.

### Infection/transmission of entomopathogens in insect farms

**Figure 2:**
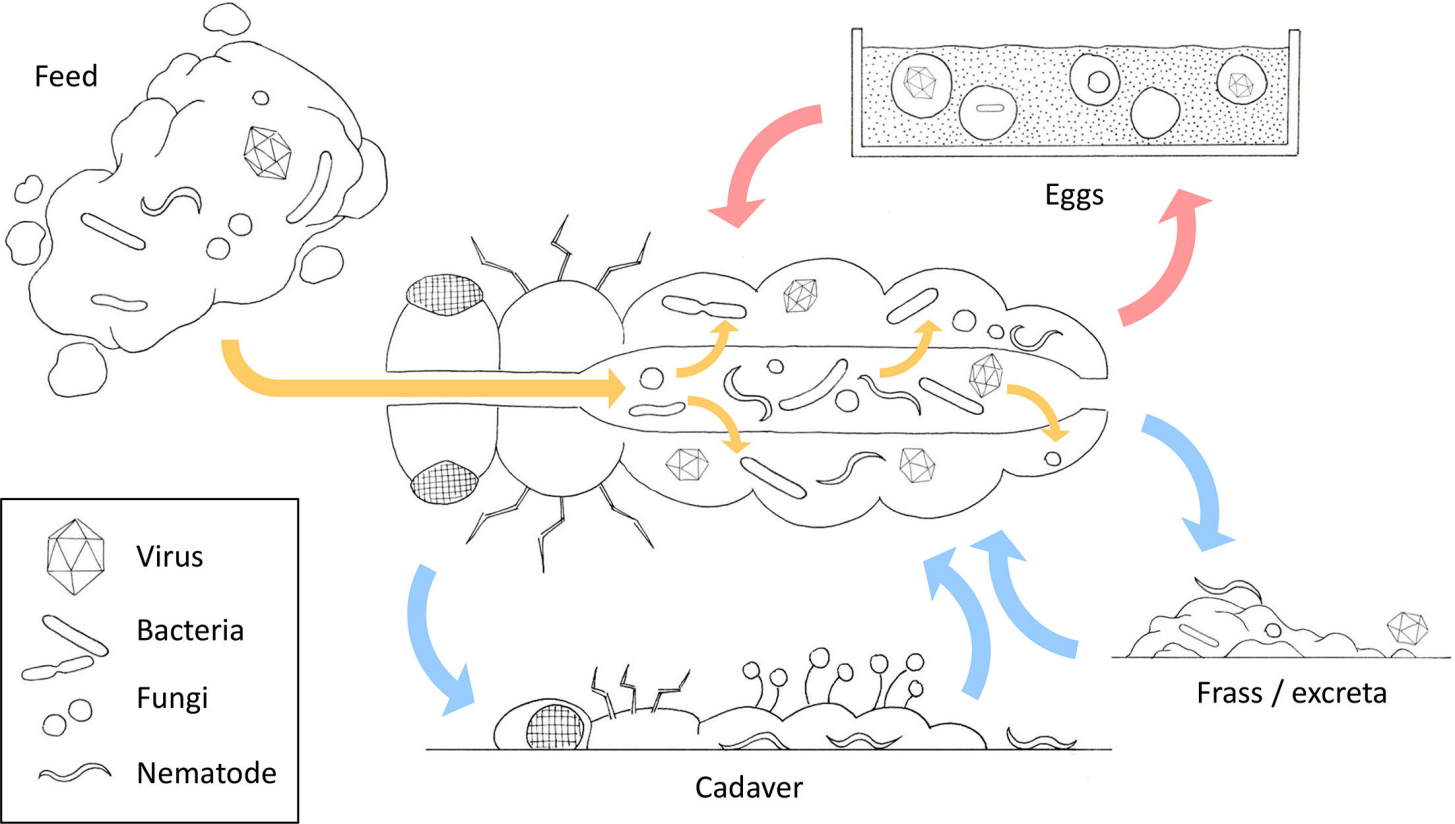
Infection routes within an edible insect farm. Pathogens enter insects orally via contaminated food, crossing the gut wall to reproduce in the haemocoel/haemolymph (orange arrows). Microbes are also present within the ‘healthy’ insect as part of the natural gut microbiota or as covert infections; however, dysbiosis (a disruption in the balance of the natural microbiota) and disease outbreaks can lead to death of the insect. Contamination of the environment and transmission to insects in the population (blue arrows) through infected frass/excreta and cadavers. Nematodes and fungi (which sporulate on the surface of cadavers) infect insects through their cuticle. Co-habitation of young and adult insects, plus transmission of covert infections to eggs which hatch to produce pre-infected nymphs/adults, can also result in vertical transmission down the generations (red arrows).

Disease-free populations of farmed insects are rare (if they exist at all), and populations are continuously exposed to fresh contamination from feed/water (infectious organisms, mycotoxins, allergens, heavy metals, etc.) and from their own excreta/frass (Figure 2) [29,52]. Insect farming is frequently intensive, characterised by mass rearing of monoculture species at artificially high densities in controlled conditions (typically high temperatures and high humidity), which is likely to promote the growth and spread of entomopathogens as well as the growth of their hosts [56]. For instance, the optimum growth temperatures of various entomopathogenic fungi (e.g. 23°C for *Beauveria brongniartii*, 25°C for *Nomureae rileyi* and *B. bassiana*, and 28°C for *M. anisopliae*) are similar to the temperatures farmed insects are reared at. Insect–entomopathogen interactions are also known to depend on (and respond rapidly to changes in) such farm-relevant environmental parameters, which affect both pathogenicity and the efficacy of the insect immune response [57–59].

Factors contributing to the development of disease in insect rearing systems are largely determined by the production conditions. Outbreaks most commonly occur when insect populations experience stresses such as temperature changes, humidity changes, dietary changes, nutrient deficiency, overcrowding, infection and exposure to toxins [60]. For instance, increased rearing density has been shown to raise cricket AdDV viral load, which is also increased by the higher temperatures required for optimal cricket production [56]. In this case, and potentially more generally, there seems to be a trade-off between production rate and risk of disease outbreak, likely mediated by insect welfare/health and its impact on immunity.

Insects do not have an adaptive immune system but can nevertheless respond to infections with humoral and cellular responses. Detection of infection via the binding of ‘pathogen-associated molecular patterns’ (PAMPs) to PAMP receptors triggers an array of responses, including the production of antimicrobial peptides (AMPs) by the insect’s ‘fat body’, parasite encapsulation and phagocytosis by haemocytes (cells in the haemolymph), apoptosis, etc. [61,62]. Infection can also trigger behavioural changes, including fever/basking to increase body temperature, which, if allowed, helps the host resist the infection [58,63–65]. Insect husbandry regimes that limit an insect’s ability to regulate its body temperature may exacerbate the progress of disease, while those that do not may permit an insect to live with a latent infection. Such interactions between insect farm conditions, host immunity and pathogen biology have considerable potential to promote or impede the emergence and spread of infectious diseases, and more research is needed in this area.

## Entomopathogen control measures

In insect farms, most outbreaks are discovered when there is already significant death or damage to infected insects, and published reports detailing such outbreaks are rare and anecdotal. Inspection, disinfection and disposal of the infected population is the usual response, resulting in welfare issues for the insects but also economic losses to the companies. The literature suggests several mitigation protocols to prevent outbreaks, such as only incorporating new individuals into a population after pathogen testing and quarantine, continuous monitoring of populations and imposing quarantine upon any mortality changes [66]. However, the application and effectiveness of these measures vary depending on the insects and pathogens in question, plus the type of rearing system [60].

### Pathogen detection

Identifying microbial pathogens in edible insects is essential for maintaining food safety and consumer confidence but could potentially also be used routinely to monitor insect population health [67]. Traditional culture-based methods of identifying fungi, oomycetes and bacteria are still widely used due to their dependability and cost-effectiveness but are slow (requiring 24–72 h to observe microbial growth) and can only be used to detect the culturable minority of microbes [68]. For large farms that require frequent monitoring across numerous batches or houses, culture alone is typically too slow and labour-intensive without automation (robotic plating and plate readers), which considerably raises capital expenses. In contrast, non-culture-based methods such as immunological assays, molecular techniques and other technologies provide greater sensitivity, specificity and speed [69]. These can, however, be cost-intensive, as the production or procurement of species-specific reagents can be costly, and commercial test availability for insect pathogens may be limited, increasing the expense of in-house testing.

Polymerase chain reaction (PCR) and quantitative PCR (qPCR) are two commonly used molecular techniques for identifying the presence of specific pathogens in food. PCR amplifies pathogen-specific DNA sequences, enabling quick detection (typically a few hours), and qPCR can quantify microbial burden [70]. Another alternative is loop-mediated isothermal amplification (LAMP), which amplifies target DNA under isothermal conditions and can be conducted without specialised equipment [71].

In-depth and efficient evaluation of food biodiversity is currently achieved by high-throughput sequencing (HTS) of samples obtained directly from the food matrices under study [49]. Barcoding approaches use PCR to amplify the conserved genetic barcodes found in all cellular organisms, which are then sequenced [72], while metagenomic analysis sequences the totality of DNA found in a sample (including viruses). Both methods are ‘untargeted’, allowing identification of all organisms present in a sample, not just culturable organisms or specifically targeted pathogens. HTS has shown that edible insect microbiomes exhibit a high level of bacterial diversity and variety, including organisms which can cause opportunistic infections in humans [49,73]. Using such methods, food spoilage bacteria have been found in mealworm larvae, as well as *Spiroplasma* spp., which have been linked to neurological illnesses in both animals and humans [49].

In addition to testing the safety of insect farm products, such technologies could be adopted by insect farms for on-site detection/diagnosis, and each has its own strengths and weaknesses (Table 2). However, running costs and the need for specialist expertise and capital investment can be significant barriers for adoption.

**Table 2:** Detection technologies and pathogen control measures which could potentially be implemented in insect farms

A: Pathogen detection	Strengths	Weaknesses	Feasibility
Microbial cultivation	Cheap, simple.	Slow. Only works for cultivatable fungi, oomycetes and bacteria.	Easily adopted for routine screening and enumeration. Defined media allow identification of targeted pathogens.
Immunological assays	Fast, targeted detection of specific pathogens.	Fairly labourious. Requires substantial technical expertise.	Allows fast detection of priority pathogens.
PCR (including qPCR and LAMP)	Fast, routine detection of specific pathogens.	Fairly labourious. Requires some technical expertise.	Good for testing presence of specific pathogens, but its sensitivity can give false positive results.
High-throughput sequencing (HTS)	Fast. Untargeted assessment of microbiome composition.	Expensive per sample, but costs continue to decrease. Requires substantial skill in molecular biology and bioinformatics methods.	Too data-rich for routine surveillance but could be used to detect dysbiosis.

B: Pathogen control	Strengths	Weaknesses	Feasibility
Stringent biosecurity protocols	Cheap and simple. Reduces pathogen entry and transmission.	Difficult to control an already established outbreak.	Easy to implement hand sanitising regimes, equipment sterilisation, waste decontamination and protocols to reduce cross-contamination.
Antimicrobials	Potent and specific activity against susceptible pathogens.	Can be expensive and many pathogens are naturally multi-drug resistant. Can trigger dysbiosis.	Could complement disinfection of an outbreak. Unsustainable: application promotes resistance in resident microbiomes.
Probiotics	Sustainable prevention of pathogen emergence.	Preventative. Cannot resolve an existing infection.	Potentially a simple and cheap way to buffer the natural microbiome against dysbiosis.
Biological control	Natural predators reproduce as they consume pathogens.	Could potentially trigger dysbiosis or emergence of resistance in pathogens. Can be pathogen specific.	Cheap and simple. Could be used to prevent infections and resolve ongoing infections. Potential to evolve to target resistant pathogens.
Antimicrobial peptides and digestive enzymes	Potent antimicrobials against a broad spectrum of pathogens.	Expensive to manufacture. Relatively labile compared with probiotics or biological control agents.	Potent, but likely to require regular/repeated administration if used as a prophylactic.
Breeding resistant insects	Insects become intrinsically resistant to pathogens	Novel pathogens are likely to emerge which can target resistant hosts.	Requires a lot of research and development. Takes a long time to replace non-resistant stock.

### Prevention and disinfection

To prevent or resolve a pathogen outbreak, several treatment options are possible (Table 2). Stringent biosecurity and hygiene protocols (such as equipment sterilisation, proper waste management and controlled access to raising units) must be adopted routinely to reduce the risk of introducing novel pathogens and to prevent the spread of endogenous or feed-associated pathogens [66,74]. However, such measures cannot completely prevent outbreaks, and effective monitoring for infections could potentially allow interventions to mitigate the severity of the outbreak.

Use of chemical treatments such as antibiotics and antifungals is a possible, but not sustainable, option for treating infected insects. AMR arises when pathogenic microbes adapt to resist the effects of drugs, and the overuse of antimicrobials in agriculture has already contributed greatly to the global AMR crisis [75]. Administering such chemicals in insect farms risks promoting the evolution of resistant strains of pathogens, which could escape to devastate insect populations and pose further dangers to animals, humans and natural ecosystems. Antibiotic treatment can also cause dysbiosis in the insect gut, making organisms more susceptible to the overgrowth of potential pathogens [76].

Insects reared for human consumption must adhere to food safety guidelines, and the use of antibiotics in insect farming raises additional concerns about chemical residues in the finished product, allergic reactions in consumers and the horizontal transfer of resistance genes down the food chain [66]. Rather than attempting to eliminate microbial pathogens, a more practical and effective approach might be to use treatments to reduce pathogenic microbial loads to levels that do not cause problematic rates of transmission (i.e. when insect growth/reproduction exceeds death/transmission rates), reducing the likelihood of resistance development while conserving beneficial microbiota [77].

### Potential treatments

One strategy to achieve this is the introduction of probiotic bacteria to modulate the gut microbiome, which can considerably lower infection susceptibility by preventing dysbiosis [73,78–80]. An extreme form of this strategy is biological control – using antagonistic/predatory organisms to specifically target pathogens. For example, the bacterial predator *Bdellovibrio bacteriovorus* has great potential as a ‘living antibiotic’ against bacterial pathogen ‘prey’ and has been used successfully to help treat *Shigella* infection in zebrafish [77,81]. Other sustainable options to hinder pathogens could include administering AMPs (including those derived from non-insects), using digestive enzymes that target pathogen outer surfaces [82], and breeding disease-resistant lineages [83].

## Perspectives

Farming insects for food is relatively new in Western cultures and is growing rapidly, manifesting an urgent need for novel disease prevention/control approaches. Particularly important priorities for research in the short-to-medium term include (i) comprehensively describing outbreaks for publication in the literature, (ii) developing a better understanding of the insect-microbiome and intramicrobiome interactions in healthy and diseased insects in farm contexts (and its impact on insect health and yield), and (iii) developing bioactive treatments to reduce pathogen burden. In addition to developing technological approaches to detect and fight pathogens, more in-depth policy and legislation are needed for insect farming, ideally at a global level. A One Health standpoint is required to ensure insect farmers are trained in best practices for disease management (as soon as possible), facility design and environmental monitoring, which are critical for long-term sustainable insect protein production.

**Summary**

The insect farming industry is growing exponentially and produces nutritional protein more sustainably than traditional agriculture. To support further responsible growth of the industry, we recommend:

- Strengthen disease surveillance and control. Intensive farming of insects is susceptible to the emergence and spread of entomopathogens, which affect food safety and reduce farm productivity.
- Characterise the microbiomes of framed insects. Even healthy insects are hosts to a wide range of potentially entomopathogenic organisms, as well as pathogens which can potentially infect humans.
- Investigate microbiome stability and disease triggers. In addition to fresh infection from contaminated feed, endogenous covert infections can become active infections and cause disease outbreaks, likely triggered by dysbiosis in the insect microbiome.
- Develop probiotic and biological control strategies. The addition of probiotics and biological control agents has the potential to suppress the growth of entomopathogens, protecting farmed insect populations from disease outbreaks.

## Open Access

This article has been published open access under our Subscribe to Open programme, made possible through the support of our subscribing institutions, learn more here: https://portlandpress.com/pages/open_access_options_and_prices#conditional

## Abbreviations

AMPs: antimicrobial peptides
AMR: antimicrobial resistance
AdDV: *Acheta domesticus* densovirus
CAGRs: compound annual growth rates
EU: European Union
PAMPs: pathogen-associated molecular patterns
PCR: polymerase chain reaction
qPCR: quantitative PCR
